# Exploring the Binding Mechanism of ADGRG2 Through Metadynamics and Biochemical Analysis

**DOI:** 10.3390/ijms26010167

**Published:** 2024-12-28

**Authors:** Chao Zhang, Ru Zhang, Yuanyuan Qi, Xin Wen, Jinpeng Sun, Peng Xiao

**Affiliations:** 1 Department of Biochemistry and Molecular Biology, School of Basic Medical Sciences, Cheeloo College of Medicine, Shandong University, Jinan 250012, China; 202020697@mail.sdu.edu.cn (C.Z.); ruzhang2000@163.com (R.Z.); yuanyuanqi1126@163.com (Y.Q.); xinwensdu@126.com (X.W.); 2 Advanced Medical Research Institute, Cheeloo College of Medicine, Shandong University, Jinan 250012, China

**Keywords:** GPCR, antagonists, metadynamics simulation, ligand binding assay, cAMP assay

## Abstract

G protein-coupled receptors (GPCRs) play essential roles in numerous physiological processes and are key targets for drug development. Among them, adhesion GPCRs (aGPCRs) stand out for their unique domain structures and diverse functions. ADGRG2 is a member of the aGPCR family and is involved in the regulation of various systems in the human body, including reproductive, nervous, cardiovascular, and endocrine systems. Investigating ADGRG2 antagonists enhances our understanding of its regulatory roles in diverse physiological processes, yet their precise mechanisms of action remain unclear. To address this, we investigated the antagonistic mechanism of ADGRG2 by examining its interactions with various antagonists, including short peptides (F601D, F601E) and small molecules (deoxycorticosterone, DOC). Using advanced metadynamics simulation, ligand binding assay and cAMP assay, we elucidated the binding modes of these antagonists. We identified five distinct F601D-ADGRG2 complex states, four F601E-ADGRG2 complex states, and three DOC-ADGRG2 complex states, which were each characterized by specific hydrogen bonds or polar interactions with their respective ligands. Although the ADGRG2 binding pocket consists of both polar and hydrophobic residues, our biochemical experiments revealed that mutations in polar amino acids significantly reduce the efficacy of the antagonists. Our results show that F601D, F601E, and DOC induce the formation of Y758^ECL2^-N775^5.32^-N860^7.46^ polar networks within ADGRG2, effectively stabilizing its inactive state. Additionally, we compared the active and inactive states of ADGRG2, highlighting the structural changes induced by antagonist-stabilized polar networks and their impact on receptor conformation. These findings provide important insights into the biology of aGPCRs and provide theoretical support for the rational design of therapeutic drugs targeting ADGRG2.

## 1. Introduction

G protein-coupled receptors (GPCRs) represent one of the most significant and diverse families of membrane receptors in the biomedical field, encompassing over 800 members that play critical roles in various physiological and pharmacological processes [1,2,3]. Among these, adhesion GPCRs (aGPCRs) form a unique subset characterized by unique structural domains and a wide range of physiological functions [4,5,6]. ADGRG2, a member of the aGPCR family, has been implicated in reproductive, neurological, cardiovascular, endocrine, and oncological processes [7,8]. Its extracellular domain contains unique structural features such as the N-terminal domain and the conserved GPCR proteolysis site (GPS) within the GPCR autoproteolysis-inducing (GAIN) domain [9,10]. Under physiological conditions, self-cleavage of the ADGRG2 GPS site produces α and β subunits. The α subunit is the extracellular region, and the β subunit is the region containing seven transmembrane (7TM) helices. The N-terminal part of the β subunit is called the Stachel sequence, which plays a key role in ADGRG2 activation [11,12]. Despite advancements in understanding ADGRG2 and its ligands, the precise molecular mechanisms underlying Stachel sequence-mediated signaling and receptor activation or antagonism remain elusive [13,14]. 

Targeting ADGRG2 with antagonists holds considerable therapeutic potential for treating diseases linked to its abnormal signaling, particularly in reproductive health and cancer [15,16]. Furthermore, studying ADGRG2 antagonists not only enhances our understanding of unique structure–function relationships but also offers valuable insights for the development of highly specific drugs. Given the critical role of ADGRG2 in various diseases, it represents an attractive target for therapeutic intervention [17,18]. However, designing GPCR antagonists is inherently challenging due to the complex membrane-bound structure and diverse conformational states of GPCR receptors [19,20]. Therefore, a deeper understanding of the GPCR antagonism mechanism can guide new drug development and promote therapeutic research on related diseases [21,22]. In our previous research, we identified endogenous agonists and antagonists of ADGRG2, such as the steroid hormone dehydroepiandrosterone (DHEA) and its sulfate (DHEAS) as agonists, and the steroid hormone deoxycorticosterone (DOC) as an antagonist [23], all of which are naturally occurring compounds. In addition, we obtained a short peptide antagonist of ADGRG2 by a series of negatively charged mutations of the newly developed agonist VPM-IP15 [8,11]. It is well documented that minor structural changes in ligands can result in opposite properties [24]. However, the binding modes of short peptide antagonists and small molecule antagonists with ADGRG2 remain largely unexplored, and the inactivation mechanisms of aGPCRs are still unclear.

To address these gaps, we employ metadynamics (MetaD) simulations [25,26], FlAsh-BRET based ligand binding assay and cAMP assay to elucidate the inactivation mechanisms of ADGRG2 and investigate the binding modes of short peptide and small molecule ligands. MetaD is an enhanced sampling simulation technique [27,28,29] that introduces harmonic bias potentials to help the system overcome higher energy barriers, exploring a broader conformational space of proteins, thereby offering more possibilities for studying complex simulation systems. This technique is particularly effective in capturing rare and complex events such as ligand binding and dissociation [30,31,32,33,34], protein-protein/membrane/nucleic acid interactions [35,36,37,38], protein folding and unfolding [39,40], GPCR activation [12,41,42,43], and allosteric modulation [44,45,46,47,48]. Ligand binding assay and cAMP assay are utilized to explore the effect of mutations on ligand affinity and the activation of downstream signaling. We aim to clarify the molecular mechanisms of ADGRG2 antagonism, focusing on its interactions with ligands. Using computational and biochemical methods, we will examine the binding modes and conformational changes caused by peptide and small molecule antagonists. This analysis will enhance our understanding of aGPCR biology and guide the design of ADGRG2-targeted drugs.

## 2. Results

### 2.1. Selection of Antagonistic Ligands for ADGRG2 in Experimental Design 

**Figure 1:**
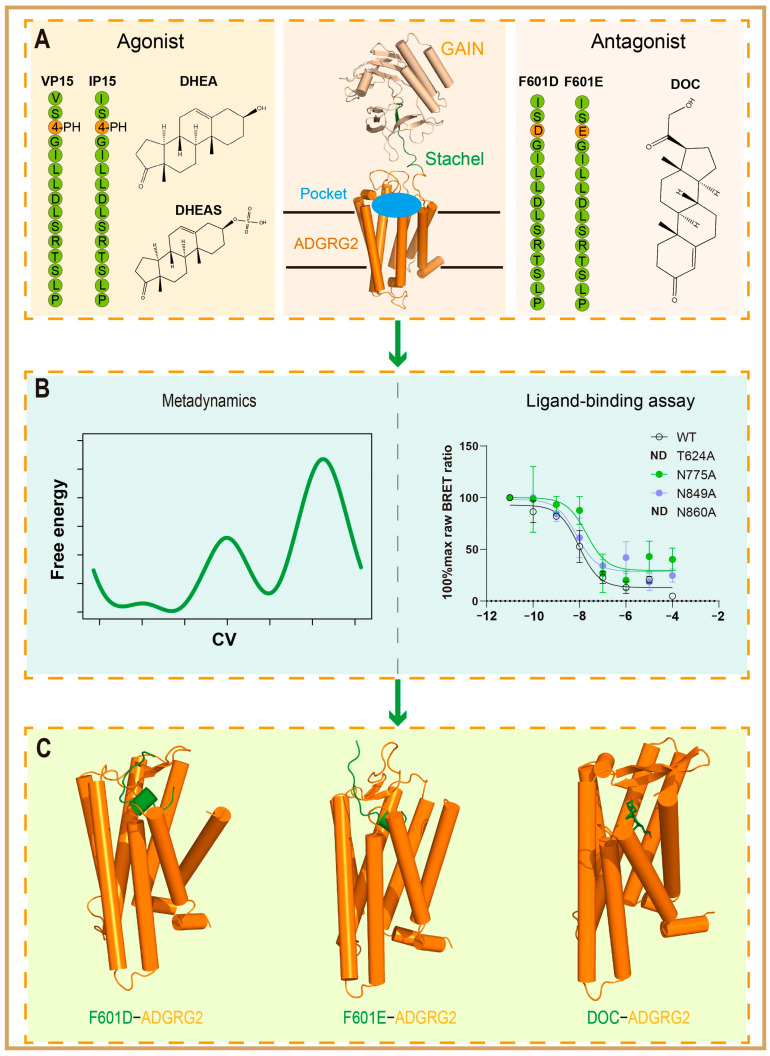
Structural and binding analysis of ADGRG2 with agonists and antagonists. (**A**) Structure of ADGRG2, highlighting key domains involved in ligand binding. Identified agonists (DHEA, DHEAS, IP15) and antagonists (DOC, F601D, F601E) are labeled. Stachel peptide sequences are presented, emphasizing mutated residues in IP15 and VP15 that enhance agonistic activity. (**B**) Schematic representation of metadynamics simulations and ligand binding assay conducted to investigate the binding mechanisms of antagonists F601D, F601E, and DOC to ADGRG2. (**C**) Structural representation of ADGRG2 in complex with antagonists F601D, F601E, and DOC, illustrating the specific binding mode of the ADGRG2-antagonists complex.The binding mode was obtained by analyzing the free energy landscape (FEL) from metadynamics simulations, extracting structural coordinates from low-energy minima defined by the root-mean-square deviations (RMSDs) of the antagonist and binding pocket residues as collective variables.

Stachel (sequence: TSFGILLDLSRTSLP, with individual residues numbered from ss01 to ss15, where “ss” denotes Stachel sequence) is a key functional element of ADGRG2, playing a critical role in regulating the conformation and function of the receptor [8]. The Stachel sequence plays a pivotal role in receptor activation by mediating ligand binding and initiating the conformational changes required for signal transduction, highlighting its biological significance [49,50,51,52,53,54]. In previous studies, the wild-type ADGRG2 Stachel sequence-derived peptidic agonist, VPM, was found to exhibit limited activation effects [8]. To enhance its activity, we introduced mutations at specific positions within the sequence for several reasons. Substituting T^ss01^ with V^ss01^, threonine (T) is a polar residue that may reduce hydrophobic interactions between the peptide and the receptor. Replacing threonine with valine (V), a hydrophobic residue, was intended to improve the interaction between the peptide and the receptor, enhancing its binding and activation efficiency. Additionally, another option is substituting F^ss03^ with 4PH^ss03^ (VPM to VPM-VP15; hereafter referred to as VP15). Phenylalanine (F) at position 3 is aromatic, but its interaction with the receptor may not be optimal for enhancing activation. By replacing F with 4-methylphenylalanine (4PH), we aimed to increase the peptide’s hydrophobicity and its interaction with the receptor, thus potentially increasing the activation effect by stabilizing the peptide–receptor complex. Further optimization resulted in VPM-IP15 (sequence: IS 4PH GILLDLSRTSLP; hereafter referred to as IP15), which replaced V^ss01^ with I^ss01^. The valine (V) at position 1 was replaced with isoleucine (I), which is also hydrophobic but may have slightly different steric properties. This substitution was designed to further optimize the peptide’s binding affinity and stability, ultimately improving its ability to activate the receptor. VPM-IP15 demonstrated superior agonistic activity (Figure 1A). We then resolved the Cryo-EM complex of ADGRG2-IP15 (PDB ID: 7WUI) and performed structural analysis and biochemical experimental verification, which provided important insights into the interaction mechanism between ADGRG2 and its ligands. The synthesis, structural analysis, and biological activity evaluation of VPM-VP15 and VPM-IP15 offered valuable clues about the interaction between ADGRG2 and its ligands. Particularly, the structural analysis of IP15 deepened our understanding of the receptor’s structure–function relationship. Building on these findings, we introduced negatively charged mutations at residue F^ss03^ (F601), leading to the discovery of two peptide antagonists, VPM-F601D (VPM-F601D refers to the peptide where phenylalanine (F) at position 601 is mutated to aspartic acid (D), abbreviated as F601D) and VPM-F601E (VPM-F601E refers to the peptide where phenylalanine (F) at position 601 is mutated to glutamic acid (E), abbreviated as F601E). These antagonists effectively stabilized ADGRG2 in its inactive state, highlighting the Stachel sequence’s crucial role in receptor regulation. In addition to Stachel sequence-derived peptides, our earlier work identified steroid hormone ligands as modulators of ADGRG2 activity. Dehydroepiandrosterone (DHEA) and its sulfate derivative (DHEAS) were characterized as endogenous agonists, while deoxycorticosterone (DOC) was identified as an endogenous antagonist [23] (Figure 1A). Thus, the antagonistic ligands F601D, F601E, and DOC were utilized as critical tools to investigate the structural changes and interaction networks that stabilize ADGRG2 in its inactive state. To elucidate the binding mechanisms and antagonistic effects of these ligands, we employed a combination of advanced computational techniques, including metadynamics simulations and biochemical assays. These approaches provided a theoretical foundation for achieving the more precise antagonistic regulation of ADGRG2. 

### 2.2. MetaD Simulation Reveals Multiple Inactive States of F601D-ADGRG2 and Their Structural Basis

**Figure 2:**
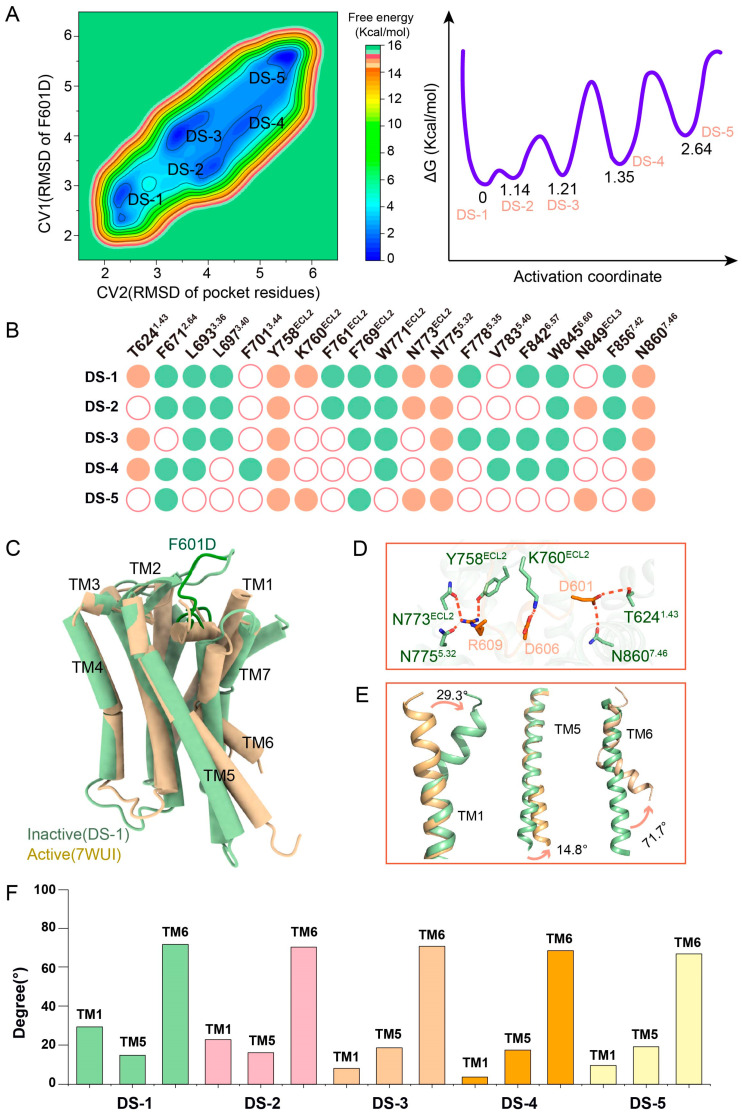
Structural and energetic characterization of F601D-ADGRG2 inactive states through metadynamics simulation. (**A**) Free energy landscape of F601D-ADGRG2 obtained from metadynamics simulation. This plot reveals the five distinct low-energy states (DS-1 to DS-5) of the F601D-ADGRG2 complex, representing different inactive conformations of the receptor; the unit of measurement is angstroms (Å). The activation coordinate represents the progression of conformational changes in ADGRG2 during antagonist-induced stabilization of inactive states. (**B**) Bar chart showing the polar interactions and hydrogen bonds formed between F601D and specific amino acids (T624^1.43^, Y758^ECL2^, K760^ECL2^, N773^ECL2^, N775^5.32^, N860^7.46^) across the five inactive states (DS-1 to DS-5). Green dots indicate hydrophobic interactions between F601D and ADGRG2,orange dots indicate hydrogen bonds or polar interactions between F601D and ADGRG2, white dots indicate no interactions between F601D and ADGRG2. This panel highlights the frequency of interactions between F601D and the residues stabilizing each state. (**C**) Structural comparison between the inactive state DS-1 of F601D-ADGRG2 and the active state ADGRG2 (PDBID: 7WUI). We focus on the displacement of key transmembrane helices (TM1, TM5, and TM6) in DS-1 relative to the active state. (**D**) Demonstration of the polar network formed by F601D and ADGRG2. In DS-1 state, D601, D606 and R609 of F601D can form a polar network with T624^1.43^, Y758^ECL2^, K760^ECL2^, N773^ECL2^, N775^5.32^ and N860^7.46^ of ADGRG2. (**E**) Detailed view of the angular shifts of TM1, TM5, and TM6 between DS-1 and the active state of ADGRG2 (PDBID: 7WUI). We quantified the extent of movement of each transmembrane helix, showing how the structure of F601D-ADGRG2 deviates from its active conformation. (**F**) Comparison of helical angles between different inactive states (DS-1–DS-5) and the active state of ADGRG2 in the F601D-ADGRG2 system. The bar graph illustrates the angular values of transmembrane helices (TMs) TM1, TM5, and TM6 across the identified inactive states (DS-1–DS-5) from metadynamics simulations and compares them to the corresponding angles in the active state structure.

To investigate the antagonistic mechanism of ADGRG2, we utilized metadynamics (MetaD) simulation. The simulations were designed with two collective variables: CV1, representing the RMSD of the antagonist (F601D, F601E, DOC), and CV2, representing the RMSD of the pocket amino acids in ADGRG2. These variables effectively captured the binding dynamics of antagonists within the receptor pocket. Based on MetaD simulation of the F601D-ADGRG2 system, we identified five distinct inactive states, which were labeled as F601D-State1 (DS-1), F601D-State2 (DS-2), F601D-State3 (DS-3), F601D-State4 (DS-4), and F601D-State5 (DS-5); among these, DS-1 emerged as the most stable state with the lowest energy (Figure 2A). In the DS-1 state, the substitution of F with D at residue 601 enables the formation of hydrogen bonds or polar interactions with key amino acids in the ADGRG2 binding pocket, including T624^1.43^, Y758^ECL2^, K760^ECL2^, N773^ECL2^, N775^5.32^, and N860^7.46^. In contrast, the other states exhibit varying interaction patterns (Figures S1–S4). By analyzing the inactive states (DS-1–DS-5) simulated by metadynamics, we found that Y758^ECL2^, Y758^ECL2^, and N860^7.46^ of ADGRG2 can form conservative polar interactions with F601D (Figure 2B). The structural model of DS-1 shows significant deviations from the active state of ADGRG2 (PDB ID: 7WUI), particularly in the angular positions of key transmembrane helices (TM1, TM5, and TM6) (Figure 2C,D). To better compare the structural differences among the various inactive states (DS-1–DS-5) identified in the F601D-ADGRG2 system, we analyzed the angular orientations of key transmembrane helices (TM1, TM5, and TM6) (Figure 2E,F and Figures S1–S4). These angular variations further support the conformational changes characteristic of transmembrane helices in the inactive states. Similar to the multiple activated states observed in GPCRs [55], we hypothesize that GPCRs can also adopt multiple inactivated states. The angular deviations observed highlight the conformational flexibility of ADGRG2 in its inactive forms. Among these, DS-1 exhibits the largest angular deviations, suggesting a substantial structural rearrangement that likely establishes a high energy barrier for transitioning to the active state. We hypothesize that the multiple inactive states of F601D-ADGRG2 arise from the ability of the Y758^ECL2^-N775^5.32^-N860^7.46^ polar network to form additional complex interactions with other polar residues, such as T624^1.43^, K760^ECL2^, N773^ECL2^, and N849^ECL3^, in different conformations. This network effectively inhibits receptor activation by stabilizing inactive states through these interactions. The observed conformational variability, particularly in TM1, TM5, and TM6, underscores the dynamic nature of ADGRG2’s inactivation. DS-1, as the most stable state, likely represents the receptor’s most energetically favorable inactive conformation. The variations across DS-1 to DS-5 suggest that ADGRG2 can adopt a spectrum of inactive states, each contributing differently to receptor regulation and ligand specificity. Metadynamics simulations further indicate that these inactive states are not simply isolated conformations but are part of the binding pathway. The free energy landscape (Figure 2A) reveals that these states represent local minima, and ligand binding facilitates transitions between these states, ultimately leading to receptor activation. This dynamic interplay between the inactive states is essential for understanding how ADGRG2 responds to different ligands and regulates its activity. 

### 2.3. MetaD Simulation Reveals Multiple Inactive States of F601E-ADGRG2 and Their Structural Basis

**Figure 3:**
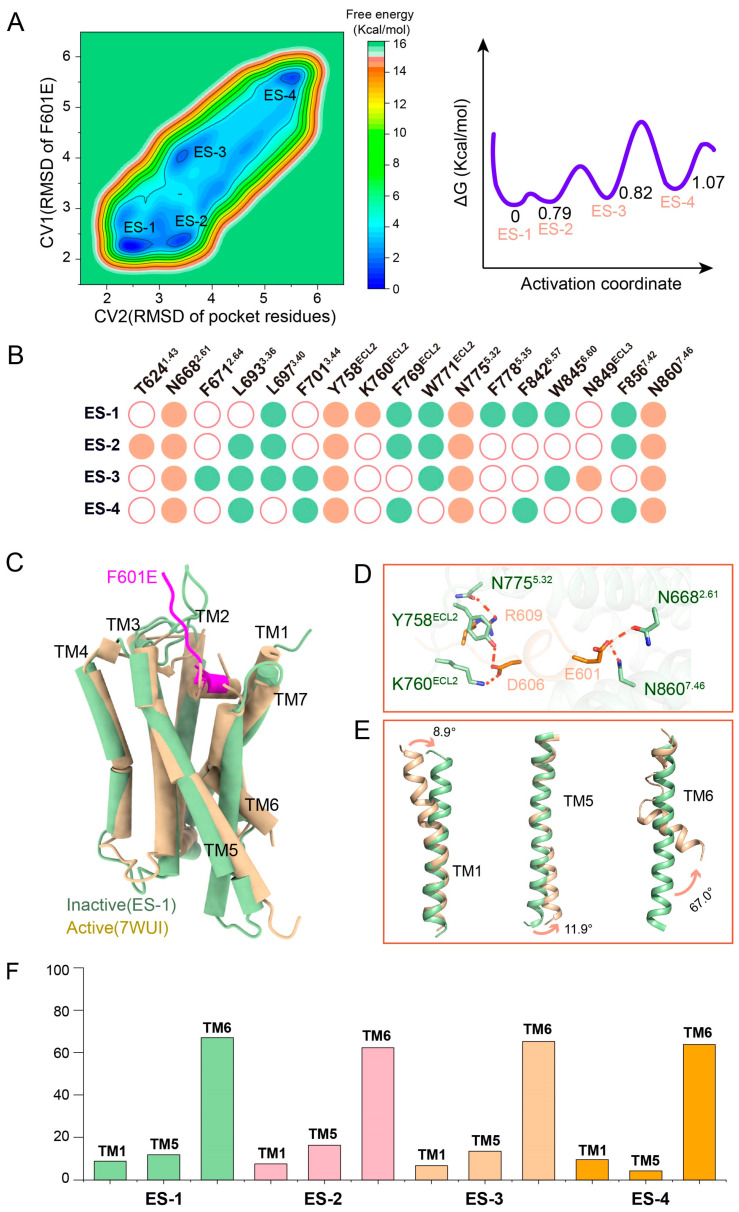
Structural and energetic analysis of F601E-ADGRG2 inactive states via metadynamics simulation. (**A**) Free energy landscape of F601E-ADGRG2 derived from metadynamics simulation, illustrating four distinct low-energy states (ES-1 to ES-4). These states represent different inactive conformations of the F601E-ADGRG2 complex; the unit of measurement is angstroms (Å). The activation coordinate represents the progression of conformational changes in ADGRG2 during antagonist-induced stabilization of inactive states. (**B**) Bar chart showing the key polar interactions and hydrogen bonds formed between F601E and specific amino acids (T624^1.43^, N668^2.61^, Y758^ECL2^, K760^ECL2^, N775^5.32^, N849^ECL3^, N860^7.46^) in each of the four inactive states (ES-1 to ES-4). Green dots indicate hydrophobic interactions between F601E and ADGRG2, Orange dots indicate hydrogen bonds or polar interactions between F601E and ADGRG2. White dots indicate no interactions between F601E and ADGRG2. The chart highlights the interactions stabilizing each conformation. (**C**) Structural comparison between the inactive state ES-1 of F601E-ADGRG2 and the active-state ADGRG2 (PDB ID: 7WUI). This panel illustrates the major conformational differences, particularly focusing on the displacement of transmembrane helices TM1, TM5, and TM6 in the ES-1 state relative to the active state. (**D**) Demonstration of the polar network formed by F601E and ADGRG2. In the ES-1 state, E601, D606 and R609 of F601E can form a polar network with N668^2.61^, Y758^ECL2^, K760^ECL2^, N775^5.32^, and N860^7.46^ of ADGRG2. (**E**) Detailed view of the angular shifts of TM1, TM5, and TM6 between DS-1 and the active state of ADGRG2 (PDBID: 7WUI). We quantified the extent of movement of each transmembrane helix, showing how the structure of F601E-ADGRG2 deviates from its active conformation. (**F**) Comparison of helical angles between different inactive states (ES-1–ES-4) and the active state of ADGRG2 in the F601E-ADGRG2 system. The bar graph illustrates the angular values of transmembrane helices (TMs) TM1, TM5, and TM6 across the identified inactive states (ES-1–ES-4) from metadynamics simulations and compares them to the corresponding angles in the active state structure.

MetaD simulations of the F601E-ADGRG2 system identified four distinct inactive states, which were designated as F601E-State1 (ES-1), F601E-State2 (ES-2), F601E-State3 (ES-3), and F601E-State4 (ES-4) (Figure 3A). Among these, ES-1 was found to be the most stable state with the lowest energy (Figure 3A). In the ES-1 conformation, F601E forms hydrogen bonds or polar interactions with residues N668^2.61^, Y758^ECL2^, K760^ECL2^, N775^5.32^, and N860^7.46^. The other states exhibit variations in their interaction patterns. By analyzing the inactive states (ES-1–ES-4) simulated by metadynamics, we found that N668^2.61^, Y758^ECL2^, N775^5.32^ and N860^7.46^ of ADGRG2 can form conservative polar interactions with F601E (Figure 3B). The ES-1 model exhibits significant differences from the active state structure of ADGRG2 (PDB ID: 7WUI) (Figure 3C,D). Specifically, angular shifts in transmembrane helices (TM1, TM5, and TM6) highlight these structural distinctions. To better compare the structural differences among the various inactive states (ES-1–ES-4) identified in the F601E-ADGRG2 system, we also analyzed the angular orientations of key transmembrane helices (TM1, TM5, and TM6) (Figure 3E,F and Figures S5–S7). Similarly, we hypothesize that the multiple inactive states of F601E-ADGRG2 arise from the N668^2.61^-Y758^ECL2^-N775^5.32^-N860^7.46^ polar network interacting with other polar residues, such as T624^1.43^, K760^ECL2^, and N849^ECL3^, in different configurations. The longer side chain of E601 allows for deeper penetration into the ADGRG2 binding pocket, enabling it to establish additional hydrogen bonds and polar interactions with key residues, particularly N668^2.61^. These enhanced interactions contribute to the formation of a more intricate polar network, which significantly stabilizes the receptor’s inactive conformation. This stabilization inhibits the structural rearrangements of the transmembrane helices, increasing the energy barrier for transitioning to the active state and thereby enhancing the antagonistic efficacy of F601E. The robust interactions mediated by E601 play a pivotal role in locking ADGRG2 in its inactive state, highlighting its importance in the receptor’s regulatory mechanism.

### 2.4. MetaD Simulation Reveals Multiple Inactive States of DOC-ADGRG2 and Their Structural Basis

**Figure 4:**
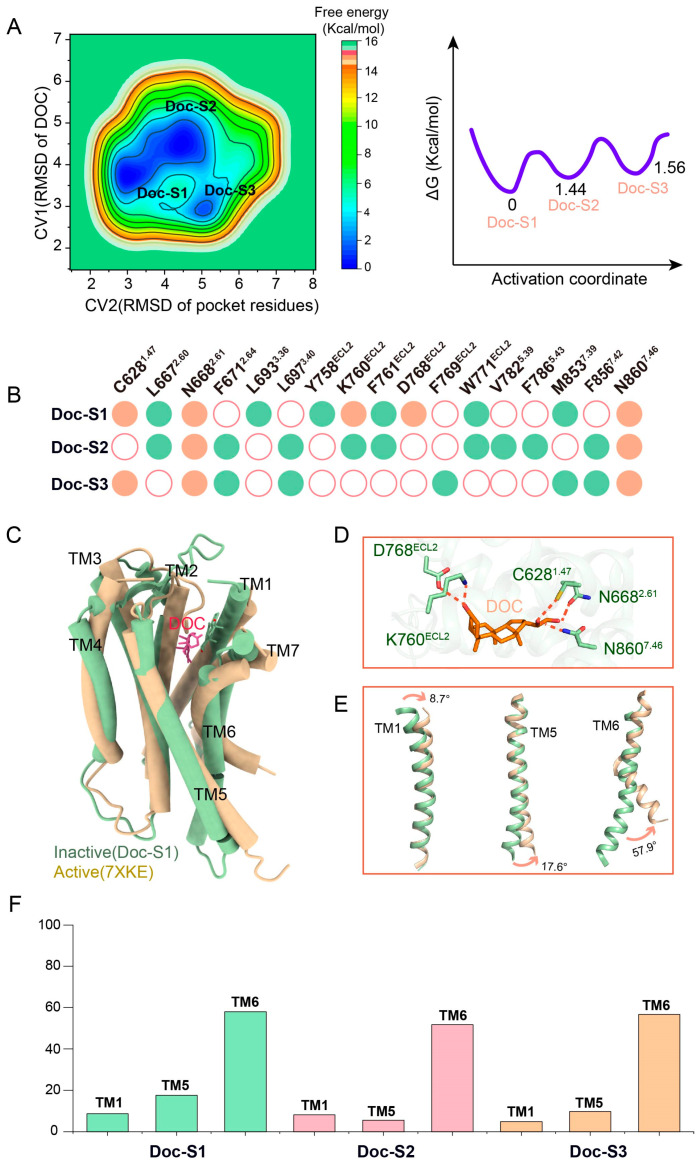
Free energy landscape and structural basis of DOC-ADGRG2 inactive states revealed by metadynamics simulation. (**A**) Free energy landscape of DOC-ADGRG2 derived from metadynamics simulation, revealing three distinct low-energy inactive states: Doc-S1, Doc-S2, and Doc-S3. These states represent different conformations of DOC-ADGRG2 in its inactive form; the unit of measurement is angstroms (Å). The activation coordinate represents the progression of conformational changes in ADGRG2 during antagonist-induced stabilization of inactive states. (**B**) Bar chart summarizing the polar interactions and hydrogen bonds between DOC and key amino acids (C628^1.47^, N668^2.61^, K760^ECL2^, D768^ECL2^, N860^7.46^) in the Doc-S1, Doc-S2 and Doc-S3 states. Green dots indicate hydrophobic interactions between DOC and ADGRG2. Orange dots indicate hydrogen bonds or polar interactions between DOC and ADGRG2. White dots indicate no interactions between DOC and ADGRG2. This panel provides a detailed breakdown of the stabilizing interactions in each state. (**C**) Structural comparison between the inactive state Doc-S1 of DOC-ADGRG2 and the active-state ADGRG2 (PDBID: 7XKE). This panel highlights the conformational differences, particularly the displacement of transmembrane helices (TM1, TM5, and TM6) in Doc-S1 relative to the active state. (**D**) Detailed analysis of the angular shifts between TM1, TM5, and TM6 in Doc-S1 compared to the active-state ADGRG2 (PDBID: 7XKE). The angular deviations are quantified, showing how these shifts contribute to maintaining the receptor in its inactive state. (**E**) Analysis of the dynamic behavior of DOC in the binding pocket based on metadynamics simulations. Despite DOC’s high mobility, polar networks formed by interactions with residues C628, K760 and D768 are sampled, which, similar to the short peptide antagonists (F601D and F601E), contribute to maintaining ADGRG2 in its inactive state. (**F**) Comparison of helical angles between different inactive states (DocS1-DocS3) and the active state of ADGRG2 in the DOC-ADGRG2 system. The bar graph illustrates the angular values of transmembrane helices (TMs) TM1, TM5, and TM6 across the identified inactive states (DocS1-DocS3) from metadynamics simulations and compares them to the corresponding angles in the active state structure.

Based on the MetaD simulation of the DOC-ADGRG2 system, we found three complex model states of DOC-ADGRG2, which were named DOC-Sate1 (Doc-S1), DOC-Sate2 (Doc-S2), and DOC-Sate3 (Doc-S3) (Figure 4A). In the Doc-S1 structure, DOC can form hydrogen bonds or polar interactions with C628^1.47^, N668^2.61^, K760^ECL2^, D768^ECL2^, and N860^7.46^. In the inactive states (Doc-S1-Doc-S3), the polar interactions that can be conserved with DOC consist of N668^2.61^ and N860^7.46^ (Figure 4B). The inactive state model of DOC-ADGRG2 (Doc-S1) obtained by enhanced sampling is significantly different from the ADGRG2 structure of the active state (PDBID 7XKE) (Figure 4C,D). Compared with the ADGRG2 of the active state (PDBID 7XKE), the angle between TM1 of Doc-S1 is 8.7°, the angle between TM5 is 17.6°, and the angle between TM6 is 57.9°. We also calculated the angular orientations of key transmembrane helices (TM1, TM5, and TM6) between various inactive states (DocS1–DocS3) identified in the DOC-ADGRG2 system (Figure 4E,F, Figures S8 and S9). Unlike peptide antagonists, DOC exhibits a more dynamic and flexible interaction profile within the ADGRG2 binding pocket. Although its interactions are weaker and less stable compared to those of peptide antagonists, DOC can still induce and stabilize multiple inactive receptor conformations by forming functional polar networks. This dynamic behavior highlights DOC’s potential as a versatile modulator of ADGRG2 activity. The flexibility of DOC’s binding allows it to sample a range of inactive conformations, offering valuable opportunities for therapeutic exploration. Specifically, optimizing DOC’s interactions within the binding pocket could enhance its stability and efficacy, making it a promising candidate for further development as a small molecule antagonist targeting ADGRG2. This flexibility may be particularly advantageous for modulating receptor activity in various disease states. 

### 2.5. Metadynamics Unveils the Role of Polar Interaction Networks in Stabilizing ADGRG2 Inactivity via Antagonist Binding

**Figure 5:**
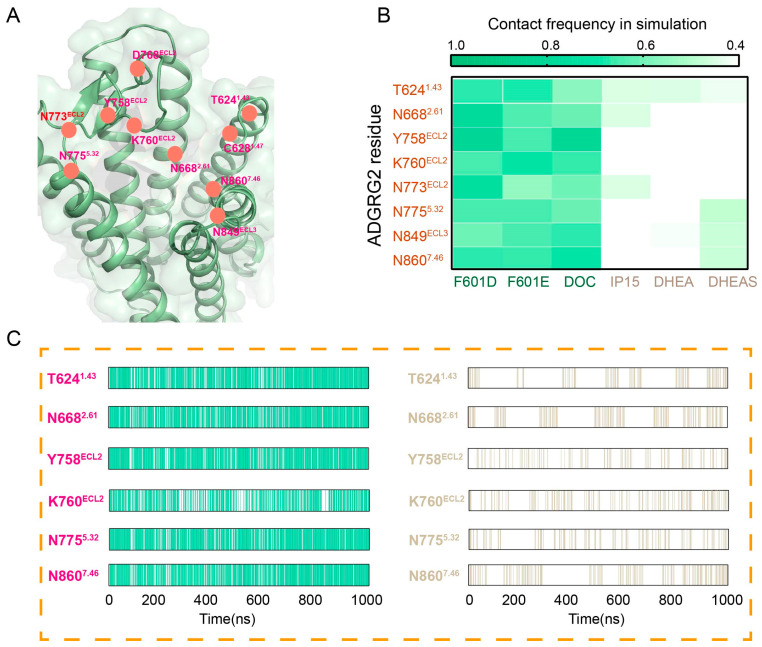
Polar residues in ADGRG2 binding pocket and their role in antagonist interactions. (**A**) Structural representation of the ADGRG2 binding pocket, highlighting the key polar amino acids (T624^1.43^, C628^1.47^, N668^2.61^, Y758^ECL2^, K760^ECL2^, N773^ECL2^, N775^5.32^, D768^ECL2^, N849^ECL3^ and N860^7.46^) that interact with antagonists (F601D, F601E, DOC). These residues are critical for stabilizing the inactive state of ADGRG2. (**B**) The frequency of interactions between the polar amino acids in the ADGRG2 pocket and antagonists (F601D, F601E, DOC) and agonists (IP15, DHEA, DHEAS) during MD simulations. This panel highlights how these residues maintain stable contact with antagonists in the inactive state but have reduced interaction frequencies with agonists. (**C**) The occupancy of hydrogen bond formation between the polar residues of ADGRG2 and ligands with the left side (magenta residues) representing interactions with antagonists and the right side (gray residues) representing interactions with agonists. The chart demonstrates that hydrogen bonding is more stable with antagonists in the inactive state than with agonists in the active state. Additionally, the occupancy shown in (**C**) is averaged over the three agonists and three antagonists, respectively, to provide a comprehensive view of the interactions.

The binding pocket of ADGRG2 contains multiple polar residues (T624^1.43^, C628^1.47^, N668^2.61^, Y758^ECL2^, K760^ECL2^, N773^ECL2^, N775^5.32^, D768^ECL2^, N849^ECL3^ and N860^7.46^), which are mainly located in the extracellular loop (ECL) region (Figure 5A). Analysis of the mutual contact frequency between antagonists and these pocket residues revealed that antagonists form stable interactions with key residues such as T624^1.43^, N668^2.61^, Y758^ECL2^, K760^ECL2^, N773^ECL2^, N775^5.32^, N849^ECL3^ and N860^7.46^ (Figure 5B). The overall contact frequency counted here covers various types of interactions, including hydrophobic interactions, van der Waals interactions, and polar interactions. To further understand the role of these polar residues, we specifically analyzed hydrogen bond contact frequencies and compared them between antagonists (F601D, F601E, DOC) and agonists (IP15, DHEA, DHEAS). The results showed a clear distinction: in the inactive state, antagonists consistently formed stable hydrogen bonds or polar interactions with these polar residues. In contrast, in the active state, these residues failed to establish stable polar interactions with agonists (Figure 5C). From this analysis, we infer that the polar groups of peptide antagonists (F601D, F601E) and the small molecule antagonist (DOC) interact with polar residues in the ADGRG2 binding pocket to form a cohesive polar interaction network. This network plays a critical role in stabilizing the receptor’s inactive conformation by inhibiting the conformational changes required for activation. This finding underscores the importance of polar interactions in ADGRG2 inhibition. The stabilization of the receptor’s inactive state through these interactions not only provides a mechanistic understanding of antagonist action but also offers a structural framework for the development of more effective antagonists in the future. Designing ligands that strengthen or exploit these polar interactions could lead to improved therapeutic strategies targeting ADGRG2.

### 2.6. Biochemical Experiments Reveal the Molecular Mechanism of ADGRG2 Antagonism

**Figure 6:**
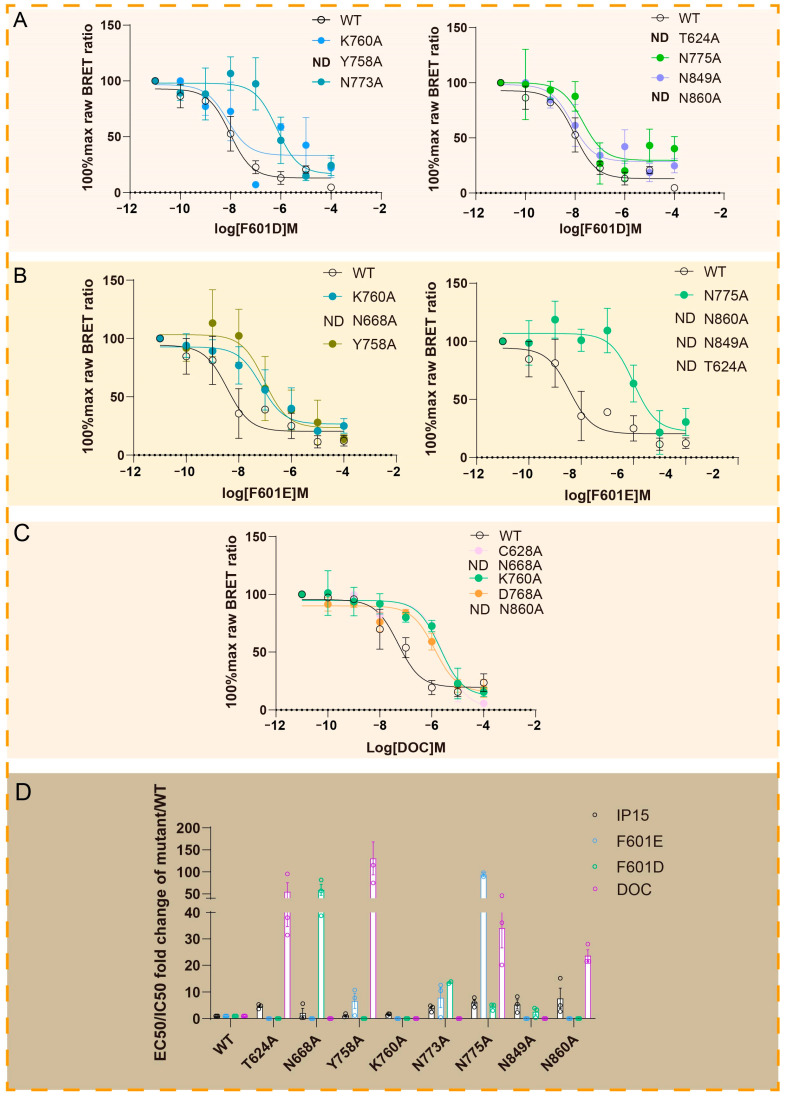
Biochemical mutagenesis experiments and cAMP assays reveal the molecular mechanism of ADGRG2 antagonism. (**A**) BRET−based binding assay showing the effects of amino acid mutations in key polar residues (T624^1.43^, Y758^ECL2^, K760^ECL2^, N773^ECL2^, N775^5.32^, N849^ECL3^, N860^7.46^) on the binding of the F601D to ADGRG2. Mutations in these residues significantly impaired F601D’s ability to bind to ADGRG2. (**B**) BRET−based binding assay illustrating the effects of amino acid mutations in key polar residue mutations (T624^1.43^, N668^2.61^, Y758^ECL2^, K760^ECL2^, N775^5.32^, N849^ECL3^, N860^7.46^) on the binding of F601E to ADGRG2. Mutations in these residues significantly impaired F601E’s ability to bind to ADGRG2. (**C**) BRET-based binding assayshowing the influence of amino acid mutations in key polar residues (C628^1.47^, N668^2.61^, K760^ECL2^, D768^ECL2^, N860^7.46^) on the binding of DOC to ADGRG2. Mutations in these residues significantly weakened DOC’s ability to bind to ADGRG2. (**D**) cAMP accumulation assay results for amino acid mutations in the presence of an agonist IP15. First, mutations were introduced to observe their effects on ADGRG2 activation with the agonist alone, followed by the addition of the antagonist to study the impact of the same mutations on antagonists binding to ADGRG2. The results confirm that the effects of these mutations are specific to the antagonists and not due to disruption of interactions between receptor residues that contribute to agonist-induced activation. Values are represented as mean ± SEM of 3 independent experiments (*n* = 3).

Based on the metadynamics simulation results of F601D-ADGRG2, F601E-ADGRG2 and DOC-ADGRG2, we revealed different binding modes of three antagonists (F601D, F601E and DOC) with ADGRG2. In the F601D-ADGRG2, F601E-ADGRG2, and DOC-ADGRG2 systems, the antagonists form hydrogen bonds or polar interactions with key residues such as T624^1.43^, Y758^ECL2^, K760^ECL2^, N773^ECL2^, N775^5.32^, N849^ECL3^, and N860^7.46^. We speculate that these polar interaction networks are important for ADGRG2 to maintain its inactive state. To verify this hypothesis, we performed amino acid mutation experiments in the three systems (Figure 6A–C), and the results showed that the mutation of the corresponding amino acids did affect the binding of antagonists to ADGRG2. To exclude the effect of amino acid mutations on ADGRG2 activation in the presence of agonists, we also tested the mutations on agonist−induced cAMP accumulation (Figure 6D). A series of biochemical mutagenesis experiments further supported the results of the metadynamics simulations; computation predicted that mutations in the identified polar residues significantly weakened the inhibitory effects of the antagonists. Mutations in these key amino acids weakened the formation of hydrogen bonds and polar interactions, which are essential for stabilizing the inactive state of ADGRG2. The combination of metadynamics data and biochemical validation highlights the importance of the polar network formed by the antagonist in inhibiting ADGRG2 activity. This comprehensive approach not only clarifies the molecular basis of ADGRG2 antagonism but also lays a theoretical foundation for future therapeutic strategies targeting this receptor family.

### 2.7. Distinct Roles of Polar and Hydrophobic Residues in the Inactive and Active States of ADGRG2

**Figure 7:**
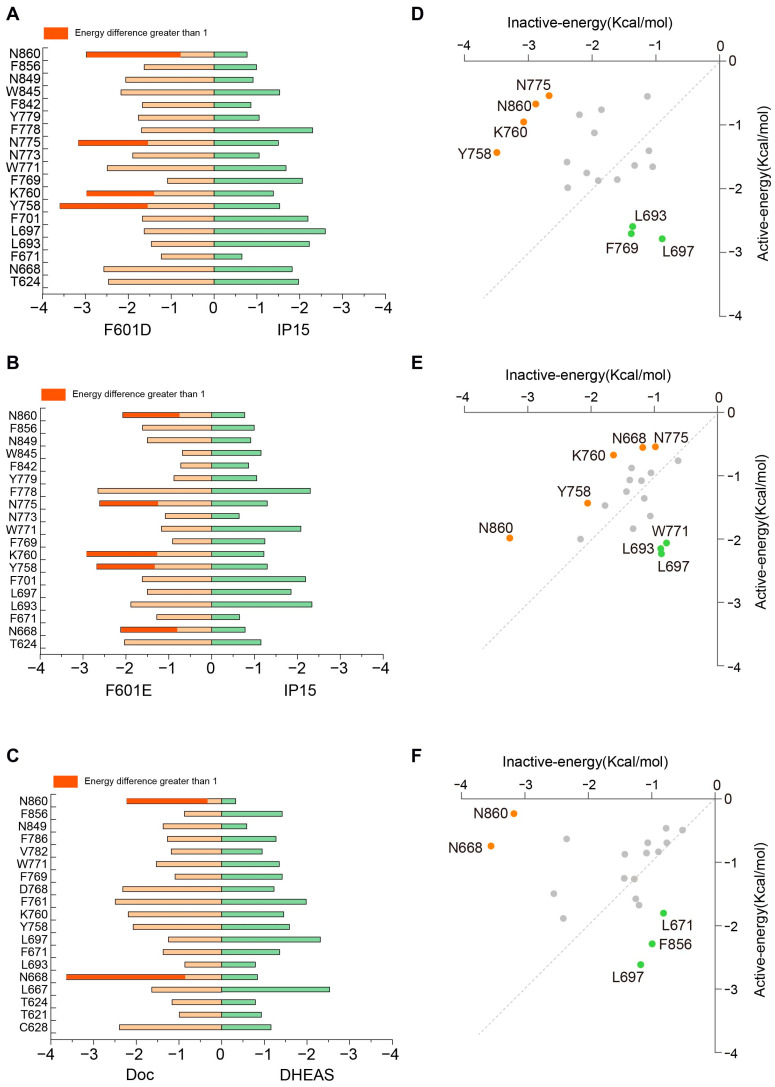
Comparison of the energy contribution of key amino acids in the inactive and active states of ADGRG2. (**A**–**C**) Comparison of energy contributions from binding site residues in ADGRG2 complexes: ADGRG2-F601D vs. ADGRG2-IP15 (**A**), ADGRG2−F601E vs. ADGRG2-IP15 (**B**), and ADGRG2-DOC vs. ADGRG2-DHEAS (**C**). White bars represent the energy contributions of key residues in the active state, light orange bars represent the energy contributions in the inactive state, and green bars indicate residues with energy differences greater than 1 kcal. (**D**–**F**) Two-dimensional (2D) plots showing energy contribution differences for binding site residues between inactive and active states in the same ADGRG2 complexes: ADGRG2-F601D vs. ADGRG2-IP15 (**D**), ADGRG2-F601E vs. ADGRG2-IP15 (**E**), and ADGRG2-DOC vs. ADGRG2-DHEAS (**F**). Orange dots represent amino acids that are more favor the inactive state, and green dots represent amino acids that are more favor the active state.

We employed the MM-PBSA method to assess the binding energies of pocket residues in various ADGRG2 complexes, including F601D-ADGRG2, F601E-ADGRG2, DOC-ADGRG2, IP15-ADGRG2 and DHEAS-ADGRG2 (Figure S10). The results revealed a clear trend: in the F601D-ADGRG2 system, polar amino acids such as Y758^ECL2^, K760^ECL2^, N775^5.32^, and N860^7.46^ primarily stabilize the inactive state of ADGRG2, whereas hydrophobic residues like L693^3.36^, L697^3.44^ and F769^ECL2^ promote the receptor’s active state(Figure 7A,D). A similar pattern emerged in the F601E-ADGRG2 system, where polar residues also play a critical role in maintaining the inactive conformation, while hydrophobic residues favor the active state (Figure 7B,E). In the DOC-ADGRG2 complex, polar residues including N668^2.61^ and N860^7.46^ are vital for preserving the inactive state, while hydrophobic residues again support the active state(Figure 7C,F). The distinct behaviors of these residues can be attributed to their roles in modulating ADGRG2’s conformational flexibility. In the inactive state, polar amino acids in the binding pocket form stable hydrogen bonds and polar interactions with antagonists such as F601D, F601E, and DOC, effectively “locking” the receptor in a conformation that prevents signal transduction. These interactions stabilize the inactive conformation by establishing a polar network that constrains the movement of key transmembrane helices, particularly TM6, which is essential for receptor activation. The integrity of this polar network ensures that ADGRG2 remains in a conformation incompatible with G-protein binding and downstream signaling. Conversely, when ADGRG2 transitions to its active state, agonists like IP15, DHEA, and DHEAS disrupt the polar interactions by favoring the engagement of hydrophobic residues. These hydrophobic interactions introduce greater flexibility in the receptor’s structure, especially in regions such as TM6 and TM5, which undergo outward movement during activation. This movement creates an opening in the receptor’s intracellular domain, facilitating G-protein coupling and signal transduction. Therefore, hydrophobic residues like L693^3.36^ and L697^3.44^ are crucial for the conformational changes required for ADGRG2 to transition from the inactive to the active state. These findings underscore the dual roles of polar and hydrophobic residues in regulating ADGRG2’s structural dynamics. Polar residues stabilize the inactive conformation by forming tight networks with antagonists, while hydrophobic residues contribute to the structural flexibility necessary for receptor activation. This study offers valuable insights into the mechanistic differences between the inactive and active states of ADGRG2, elucidating the distinct roles of polar and hydrophobic interactions in governing receptor function.

## 3. Discussion

In this study, we investigated the binding mechanisms and conformational states of ADGRG2 by analyzing its interactions with various ligands, including short peptide antagonists (F601D, F601E), small molecule antagonists (DOC), and agonists (IP15, DHEA, DHEAS). Our results, derived from MM-PBSA calculations and biochemical mutagenesis experiments, revealed the distinct roles that polar and hydrophobic residues play in stabilizing the inactive and active states of ADGRG2. Specifically, polar residues such as T624^1.43^, N668^2.61^, Y758^ECL2^, K773^ECL2^, K775^5.32^, and N860^7.46^ were critical in stabilizing the inactive state of ADGRG2 by forming strong hydrogen bonds and polar interactions with antagonists. This polar network effectively “locks” ADGRG2 in an inactive conformation, preventing signal transduction by constraining the flexibility of key transmembrane helices (TM5 and TM6). The stabilization of these helices ensures that the receptor remains in a conformation that is incompatible with G-protein binding and downstream signaling. In particular, F601D, F601E, and DOC each formed stable polar interactions that maintained this inactive state. Our biochemical mutagenesis experiments further supported this conclusion, as mutations in key polar residues such as T624^1.43^, N668^2.61^, Y758^ECL2^, K773^ECL2^, K775^5.32^, and N860^7.46^ weakened the inhibitory effects of the antagonists. The changes observed in different inactive states (DS-1–DS-5, DS-1–DS-4, DocS1-DocS3) show that ADGRG2 can adopt a spectrum of distinct inactive conformations. These conformations correspond to local minima in the free energy landscape (Figure 2A, Figure 3A and Figure 4A) and represent different sub-states within the broader inactive conformational space of the receptor. Each of these sub-states contributes differently to receptor regulation and ligand specificity, as evidenced by the structural and energetic features revealed through metadynamics simulations.

In contrast, agonists such as IP15, DHEA, and DHEAS preferentially interacted with hydrophobic residues, including L693^3.36^, L697^3.44^, F769^ECL2^ and W771^ECL2^, which facilitated the conformational changes required for receptor activation. These hydrophobic interactions promoted the outward movement of TM5 and TM6, creating an intracellular opening that allowed G-protein coupling and initiated signal transduction. The disruption of the stabilizing polar network by agonists was essential for the transition from the inactive to the active state, highlighting the importance of hydrophobic residues in driving the structural flexibility required for receptor activation.

Our findings demonstrate that ADGRG2’s functional states are governed by the dual roles of polar and hydrophobic interactions. Polar residues stabilize the inactive conformation by limiting receptor flexibility, while hydrophobic residues promote the conformational dynamics necessary for activation. This understanding of ADGRG2’s mechanisms offers valuable insights for designing selective ligands. By targeting specific polar or hydrophobic interactions within the ADGRG2 binding pocket, it may be possible to develop more precise antagonists or agonists. This could have significant therapeutic benefits for diseases linked to ADGRG2 dysfunction, such as reproductive disorders and cancers. Designing ligands that either strengthen polar interactions to keep ADGRG2 inactive, or enhance hydrophobic interactions to activate it, could lead to better treatments. In conclusion, our combined computational and experimental analysis reveals how ADGRG2 transitions between inactive and active states, highlighting key interactions that could guide future therapeutic strategies.

The changes observed in different inactive states (DS-1–DS-5, DS-1–DS-4, DocS1–DocS3) show that ADGRG2 can adopt a spectrum of distinct inactive conformations. These conformations correspond to local minima in the free energy landscape (Figure 2A, Figure 3A and Figure 4A) and represent different sub-states within the broader inactive conformational space of the receptor. Each of these sub-states contributes differently to receptor regulation and ligand specificity, as evidenced by the structural and energetic features revealed through metadynamics simulations.

## 4. Materials and Methods

### 4.1. Unbiased Molecular Dynamics Simulation

To explore the stability and binding energy of the F601D-ADGRG2, F601E-ADGRG2, IP15-ADGRG2, DOC-ADGRG2, DHEA-ADGRG2 and DHEAS-ADGRG2 systems, unbiased molecular dynamics (MD) simulations were employed. For the simulation of DHEA-ADGRG2, its input structure was derived from cryo-electron microscopy data (PDBID 7XKE). In contrast, the initial simulation structures for F601D-ADGRG2, F601E-ADGRG2, and IP15-ADGRG2 were based on the model with PDBID 7WUI. On this basis, we first completed the Stachel peptide. Subsequently, using Pymol software (version 3.1), we mutated the phenylalanine (F) at position 601 to aspartate (D) or glutamic acid (E) to generate the corresponding mutant structures. As for the simulations of DOC-ADGRG2 and DHEAS-ADGRG2, their input structures were obtained through molecular docking. We used Auto Dock v.4.2.6 software to perform the molecular docking of DOC and DHEAS. The docking box size was set to 60 × 60 × 60 Å^3^, with a grid spacing of 0.375 Å, and the centroid of DOC or DHEAS was designated as the docking center. Through this method, we generated 100 docking conformations and selected the one with the best affinity for subsequent molecular dynamics simulations. The MD input files were generated via the CHARMM-GUI website [56]. The CHARMM36m force field [57] was used for F601D-ADGRG2, F601E-ADGRG2, IP15-ADGRG2,DOC-ADGRG2,DHEA-ADGRG2 and DHEAS-ADGRG2, lipids, ions, and the TIP3P model water molecules, as described by our previous studies [2,12]. The complex structures were embedded into pre-equilibrated and periodic 1-palmitoyl-2-oleoyl-sn-glycero-3-phosphocholine (POPC) membrane structures. Monte Carlo methods were used to add 0.15 M NaCl to neutralize the charge of the F601D-ADGRG2, F601E-ADGRG2, DOC-ADGRG2, DHEA-ADGRG2, and DHEAS-ADGRG2 systems. Based on membrane orientation calculations from the Orientations of Proteins in Membranes (OPM) database [58], the systems were solvated in a periodic hexagonal TIP3P water box with dimensions of approximately 180 Å × 180 Å × 200 Å. Using the gmx mdrun module from Gromacs 2019.6 [59], energy minimization was initially performed for 10,000 steps. The systems were then heated from 0 K to 310 K under the NVT ensemble (constant number of particles, volume, and temperature) for 1,000 ps. Following this, production simulations were conducted under the NPT ensemble (constant number of particles, pressure, and temperature) with harmonic restraints of 10.0 kcal mol^−1^ Å^−2^ at 1 atm pressure for another 1000 ps. For the production simulations, a total of 2000 ns was carried out using Gromacs 2019.6 under NVT and NPT conditions with a time step of 2 fs. Long-range electrostatic interactions were calculated using the particle-mesh Ewald method with a 12 Å cutoff, and the SHAKE algorithm was applied to constrain bonds involving hydrogen atoms at each 2fs integration step. All analyses were performed using Gromacs 2019.6 and VMD software (version 1.9.3) [60].

### 4.2. Binding Free Energy Analysis

The g_mmpbsa package (version 1.6) [61] was used to perform the binding free energy between residues in the F601D, F601E, IP15, DOC, DHEAS and ADGRG2 by the molecular mechanics Poisson–Boltzmann surface area method (MM–PBSA). The binding energy calculation includes three energy items: (1) potential energy in vacuum; (2) polar solvation energy; and (3) non-polar solvation energy. In this study, we extracted the last 500 ns trajectories of complex for calculation, and these trajectories were considered to be an equilibrium part of the trajectory. A solvent probe radius of 0.14 nm was used to determine the solvation energy. The solvent dielectric constant and solute dielectric constant were set to 80 and 2, respectively. The Python script MmPbSaStat.py was used for the MM–PBSA calculation, and MmPbSaDecomp.py was used to calculate individual contributions of different residues in ADGRG2.

### 4.3. Metadynamics Simulation

The binding modes of F601D, F601E, and DOC with ADGRG2 were investigated using metadynamics simulations. Gromacs 2019.6 and Plumed 2.7.1 [62] were employed for this enhanced sampling technique. Gaussian hills were added every 500 steps to bias the potential energy landscape, with an initial height of 10 kJ/mol and a width of 1000 kJ/mol, controlled by a bias factor of 10. Two collective variables (CV1 and CV2) were used to guide the metadynamics simulations of the antagonists with ADGRG2. CV1 represents the RMSD of the antagonist, which is crucial for its internal movement, while CV2 represents the RMSD of the pocket residues of ADGRG2, which influence the binding of the antagonist to the receptor.

### 4.4. Ligand Binding Assay

HEK293 cells were transiently transfected with plasmids for Nluc-ADGRG2 and its various mutants (ADGRG2-T624A, ADGRG2-C628A, ADGRG2-N668A, ADGRG2-Y758A, ADGRG2-K760A, ADGRG2-D768A, ADGRG2-N773A, ADGRG2-N775A, ADGRG2-N849A, ADGRG2-N860A). The cells were plated at a density of 3 × 10^4^ per well in a black 96-well plate and cultured for 48 h at 37 °C with 5% CO_2_. To assess saturation binding, increasing concentrations of FITC-labeled IP15 (IP15–FITC) were added, alongside or without 300 μM of unlabeled IP15, for a 40-min incubation at 37 °C. For competitive binding assays, cells were treated with 50 μM IP15–FITC and varying concentrations (10^−9^ to 10^−4^ M) of competing peptides in HBSS buffer (containing 25 mM HEPES, 10 mM glucose, 146 mM NaCl, 5 mM KCl, 1 mM MgSO_4_, 2 mM sodium pyruvate, and 1.3 mM CaCl_2_) for 40 min at 37 °C. Coelenterazine H (final concentration of 5 μM, Promega) was added just before measuring BRET signals using a Mithras LB 940 Multimode Microplate Reader. Light emissions were recorded at 460 nm (80 nm bandpass) and 535 nm (60 nm bandpass) for IP15–FITC, and the raw BRET ratio was calculated as the 535 nm emission divided by the 460 nm emission.

### 4.5. cAMP Assay

To assess the cAMP accumulation induced by receptor activation, HEK293 cells were transfected with plasmids for wild-type ADGRG2 or its mutants (ADGRG2-T624A, ADGRG2-C628A, ADGRG2-N668A, ADGRG2-Y758A, ADGRG2-K760A, ADGRG2-D768A, ADGRG2-N773A, ADGRG2-N775A, ADGRG2-N849A, ADGRG2-N860A) alongside the GloSensor plasmid or an empty vector (pcDNA3.1). Following a 24-h incubation at 37 °C with 5% CO_2_, the transfected cells were plated at a density of 4 × 10^4^ per well in 96-well plates and incubated for an additional 24 h. The cells were then pre-treated with serum-free medium containing 5% (*v*/*v*) GloSensor cAMP reagent stock solution for 2 h. cAMP signals induced by IP15 were measured immediately after adding the agonists. Luminescence was recorded for at least 20 min per well, and the data were analyzed using sigmoidal dose–response functions in GraphPad Prism v.9.0.

## Data Availability

The original contributions presented in this study are included in the article/Supplementary Material, and further inquiries can be directed to the corresponding author.

## Supplementary Materials

The following supporting information can be downloaded at https://www.mdpi.com/article/10.3390/ijms26010167/s1.
