# Supplementary material for: Exploring the Binding Mechanism of ADGRG2 Through Metadynamics and Biochemical Analysis
Source: Int J Mol Sci. 2024 Dec 28;26(1):167. doi: 10.3390/ijms26010167 (PMC11719512; doi:10.3390/ijms26010167)
Supplement: Supplementary file 1 [file ijms-26-00167-s001.zip › ijms-3362958-supplementary.pdf]

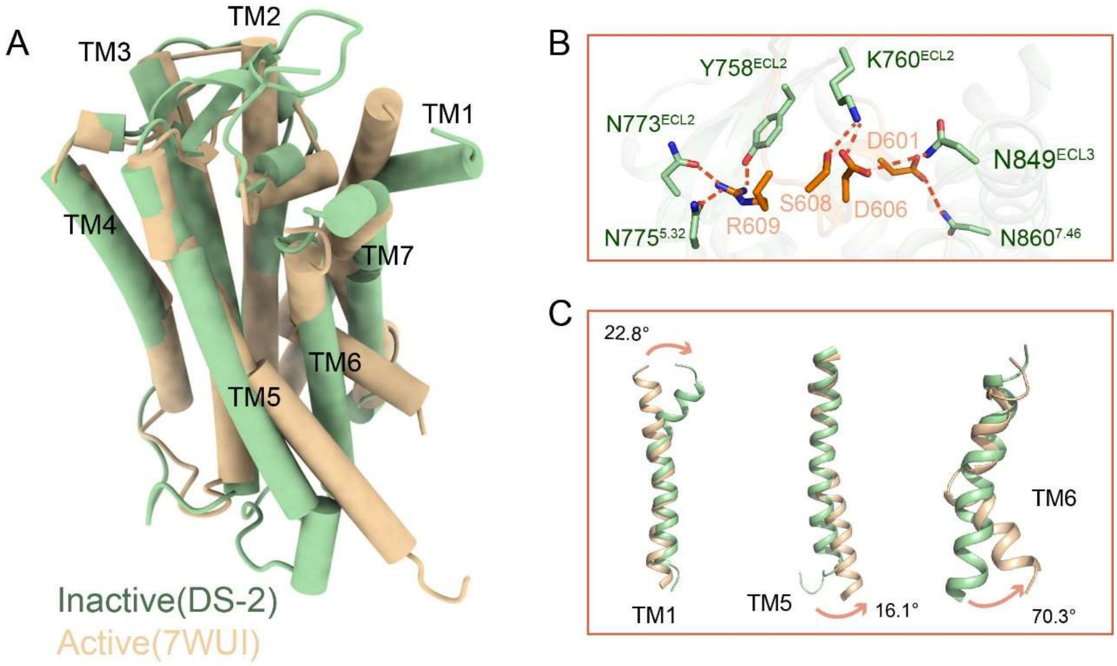

Figure S1. DS-2 of F601D-ADGRG2 obtained by metadynamics simulation. (**A**) Structural comparison between the inactive state DS-2 of F601D-ADGRG2 and the active state ADGRG2 (PDBID: 7WUI). We focus on the displacement of key transmembrane helices (TM1, TM5, and TM6) in DS-2 relative to the active state. (**B**) Demonstration of the polar network formed by F601D and ADGRG2. In DS-2 state, D601, D606 and R609 of F601D can form a polar network with Y758^{ECL2}, N775$^{5.32}$, N849^{ECL3}, N860$^{7.46}$ of ADGRG2. (**C**) Detailed view of the angular shifts of TM1, TM5, and TM6 between DS-2 and the active state of ADGRG2 (PDBID: 7WUI). We quantified the extent of movement of each transmembrane helix, showing how the structure of F601D-ADGRG2 deviates from its active conformation.

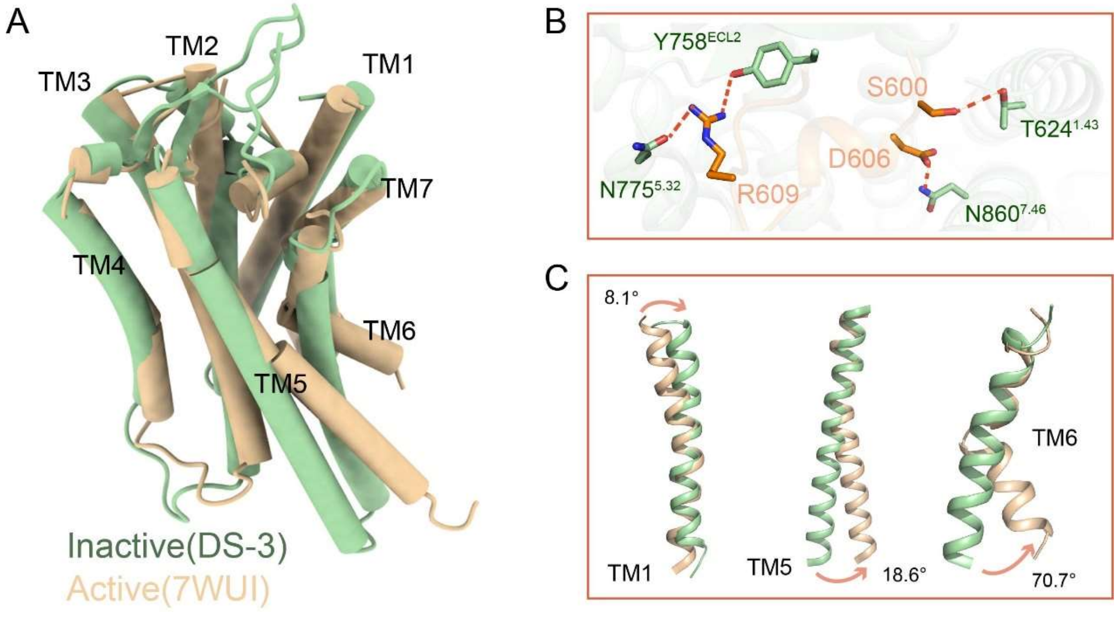

Figure S2. DS-3 of F601D-ADGRG2 obtained by metadynamics simulation. (**A**) Structural comparison between the inactive state DS-3 of F601D-ADGRG2 and the active state ADGRG2 (PDBID: 7WUI). We focus on the displacement of key transmembrane helices (TM1, TM5, and TM6) in DS-3 relative to the active state. (**B**) Demonstration of the polar network formed by F601D and ADGRG2. In DS-3 state, S600, D606 and R609 of F601D can form a polar network with T624$^{1.43}$, Y758^{ECL2},

N775$^{5.32}$, N860$^{7.46}$ of ADGRG2. (**C**) Detailed view of the angular shifts of TM1, TM5, and TM6 between DS-3 and the active state of ADGRG2 (PDBID: 7WUI). We quantified the extent of movement of each transmembrane helix, showing how the structure of F601D-ADGRG2 deviates from its active conformation.

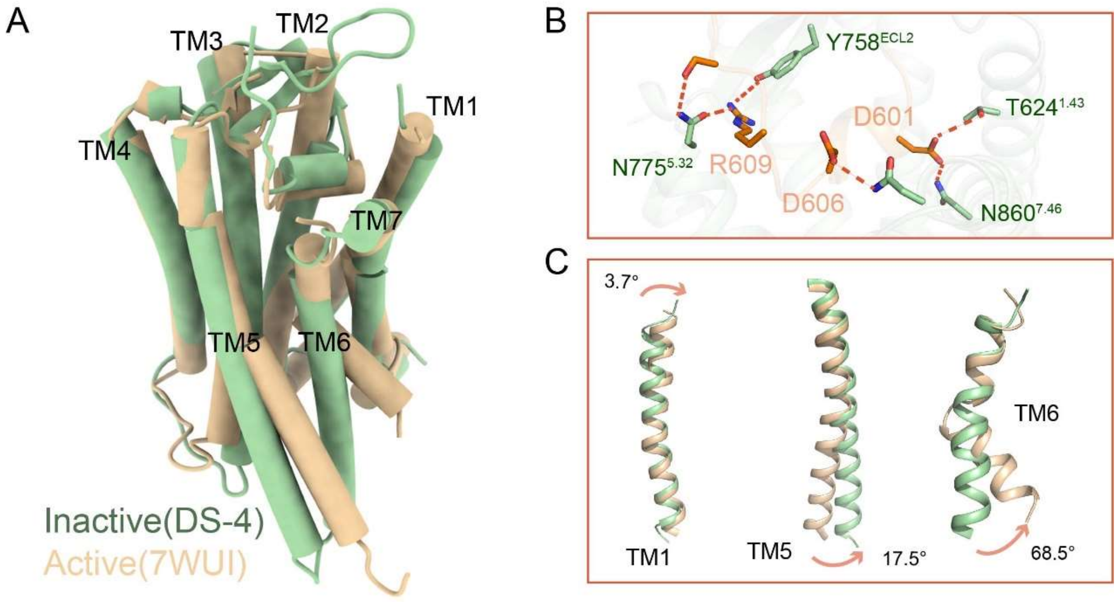

Figure S3. DS-4 of F601D-ADGRG2 obtained by metadynamics simulation. (**A**) Structural comparison between the inactive state DS-4 of F601D-ADGRG2 and the active state ADGRG2 (PDBID: 7WUI). We focus on the displacement of key transmembrane helices (TM1, TM5, and TM6) in DS-4 relative to the active state. (**B**) Demonstration of the polar network formed by F601D and ADGRG2. In DS-4 state, D601, D606 and R609 of F601D can form a polar network with T624$^{1.43}$, Y758^{ECL2}, N775$^{5.32}$, N860$^{7.46}$ of ADGRG2. (**C**) Detailed view of the angular shifts of TM1, TM5, and TM6 between DS-4 and the active state of ADGRG2 (PDBID: 7WUI). We quantified the extent of movement of each transmembrane helix, showing how the structure of F601D-ADGRG2 deviates from its active conformation.

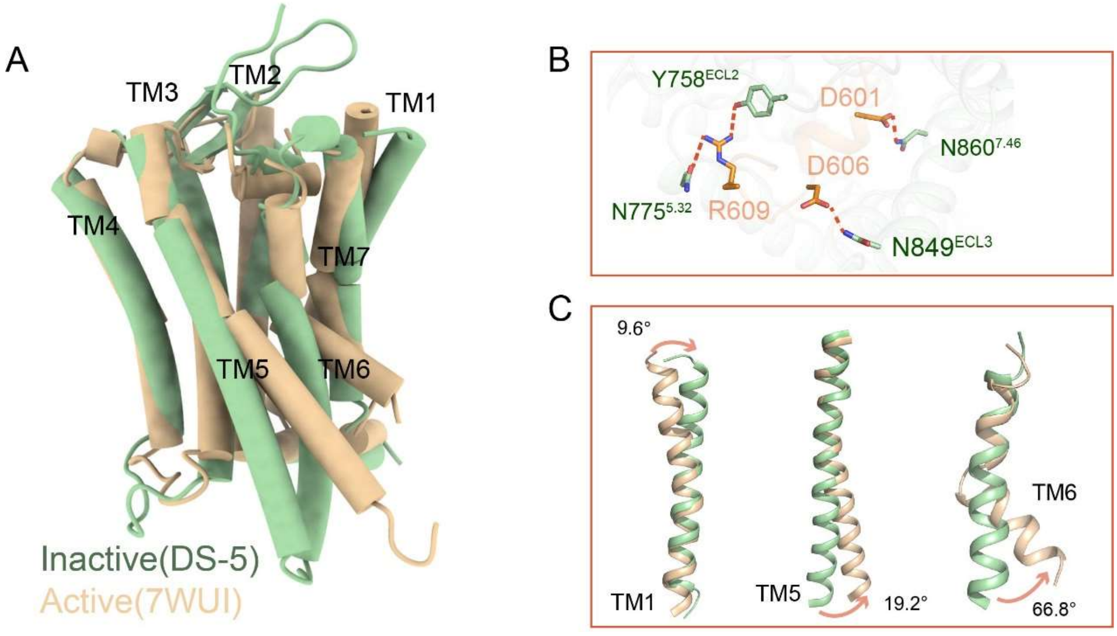

Figure S4. DS-5 of F601D-ADGRG2 obtained by metadynamics simulation. (**A**) Structural comparison between the inactive state DS-5 of F601D-ADGRG2 and the active state ADGRG2 (PDBID:

7WUI). We focus on the displacement of key transmembrane helices (TM1, TM5, and TM6) in DS-5 relative to the active state. (**B**) Demonstration of the polar network formed by F601D and ADGRG2. In DS-5 state, D601, D606, and R609 of F601D can form a polar network with T624$^{1.43}$, Y758^{ECL2}, N775$^{5.32}$, N860$^{7.46}$ of ADGRG2. (**C**) Detailed view of the angular shifts of TM1, TM5, and TM6 between DS-5 and the active state of ADGRG2 (PDBID: 7WUI). We quantified the extent of movement of each transmembrane helix, showing how the structure of F601D-ADGRG2 deviates from its active conformation.

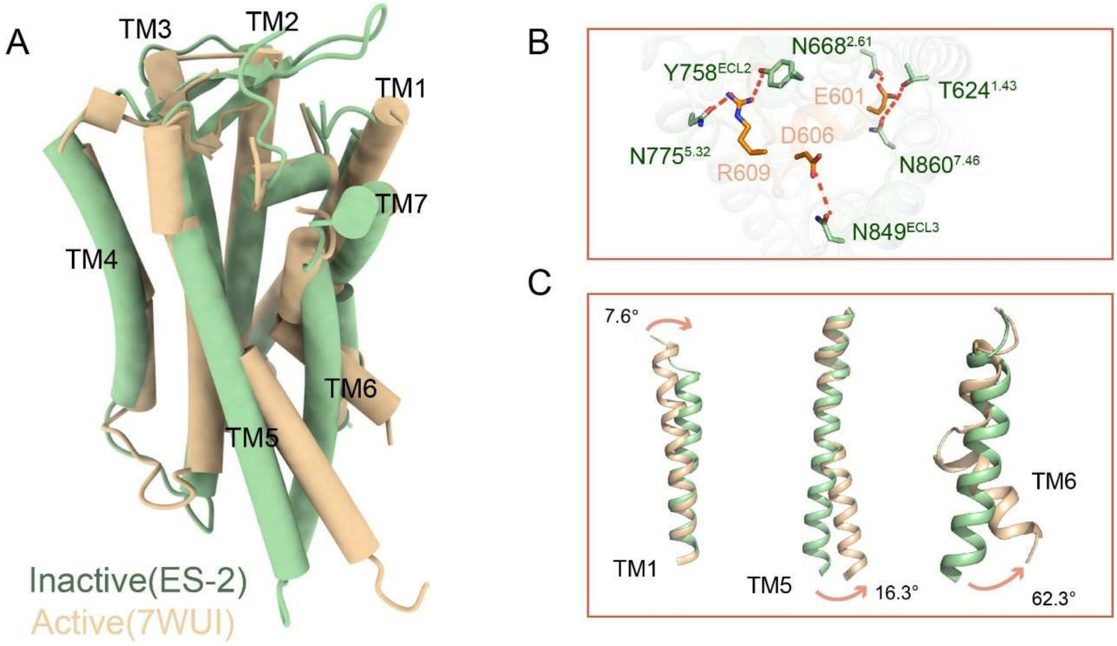

Figure S5. ES-2 of F601E-ADGRG2 obtained by metadynamics simulation. (**A**) Structural comparison between the inactive state ES-2 of F601E-ADGRG2 and the active state ADGRG2 (PDBID: 7WUI). We focus on the displacement of key transmembrane helices (TM1, TM5, and TM6) in ES-2 relative to the active state. (**B**) Demonstration of the polar network formed by F601E and ADGRG2. In ES-2 state, E601, D606, and R609 of F601E can form a polar network with T624$^{1.43}$, N667$^{2.61}$, Y758 ^{ECL2}, N775$^{5.32}$, N849^{ECL3}, N860$^{7.46}$ of ADGRG2. (**C**) Detailed view of the angular shifts of TM1, TM5, and TM6 between ES-2 and the active state of ADGRG2 (PDBID: 7WUI). We quantified the extent of movement of each transmembrane helix, showing how the structure of F601E-ADGRG2 deviates from its active conformation.

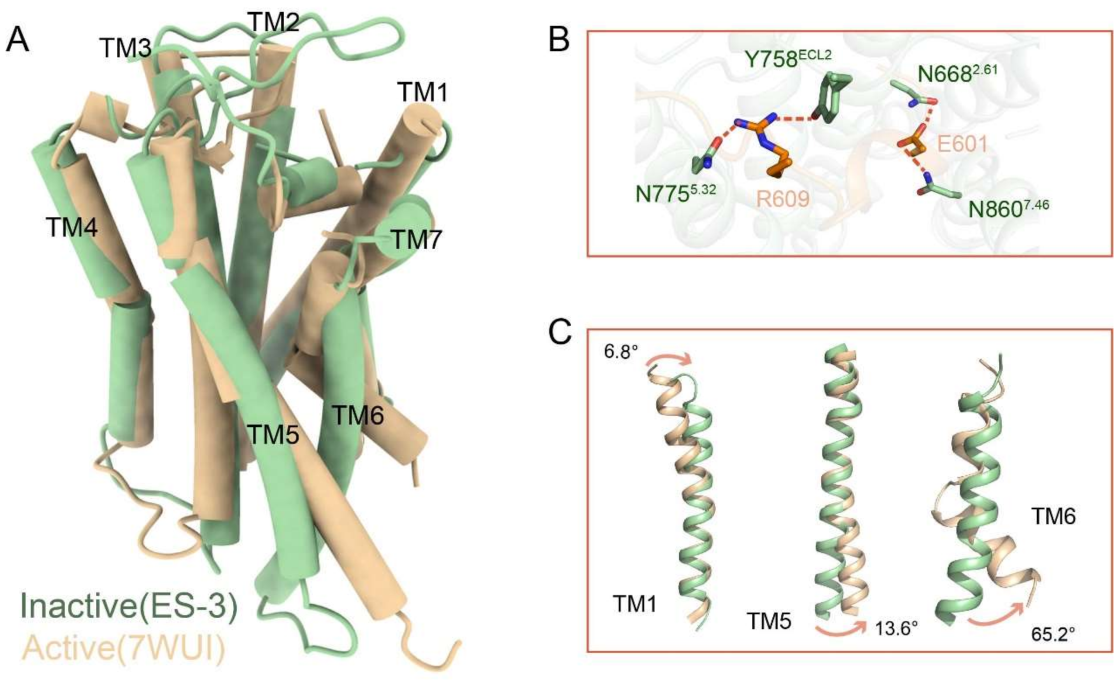

Figure S6. ES-3 of F601E-ADGRG2 obtained by metadynamics simulation. (**A**) Structural comparison between the inactive state ES-3 of F601E-ADGRG2 and the active state ADGRG2 (PDBID: 7WUI). We focus on the displacement of key transmembrane helices (TM1, TM5, and TM6) in ES-3 relative to the active state. (**B**) Demonstration of the polar network formed by F601E and ADGRG2. In ES-3 state, E601, R609 of F601E can form a polar network with N668$^{2.61}$, Y758^{ECL2}, N775$^{5.32}$, N860$^{7.46}$ of ADGRG2. (**C**) Detailed view of the angular shifts of TM1, TM5, and TM6 between ES-3 and the active state of ADGRG2 (PDBID: 7WUI). We quantified the extent of movement of each transmembrane helix, showing how the structure of F601E-ADGRG2 deviates from its active conformation.

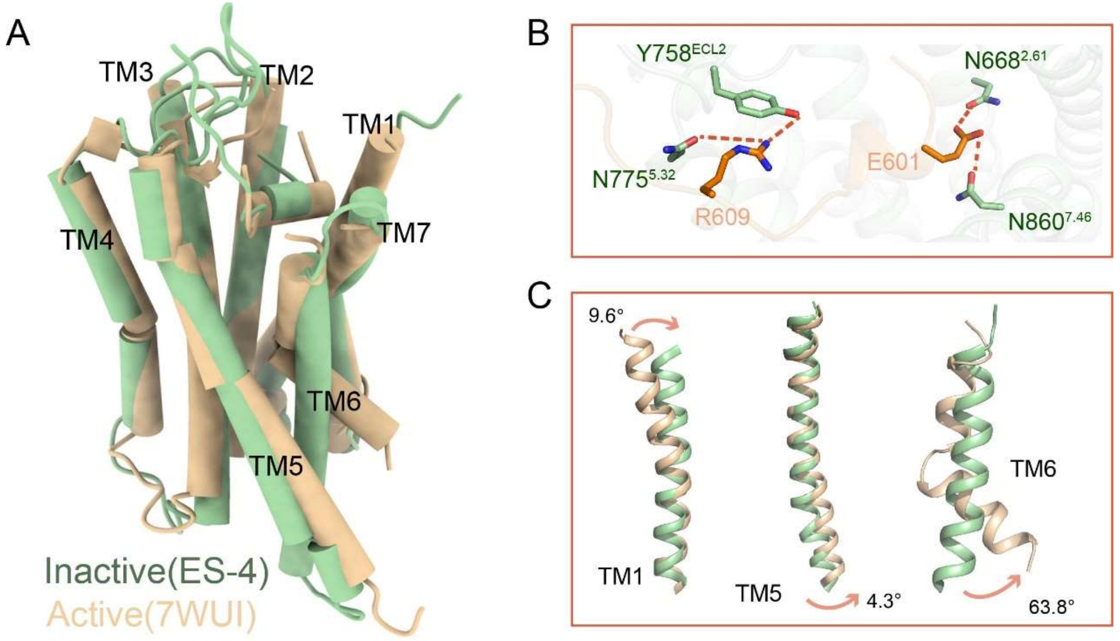

Figure S7. ES-4 of F601E-ADGRG2 obtained by metadynamics simulation. (**A**) Structural comparison between the inactive state ES-4 of F601E-ADGRG2 and the active state ADGRG2 (PDBID: 7WUI). We focus on the displacement of key transmembrane helices (TM1, TM5, and TM6) in ES-4 relative to the active state. (**B**) Demonstration of the polar network formed by F601E and ADGRG2. In ES-4 state, E601, R609 of F601E can form a polar network with N668$^{2.61}$, Y758^{ECL2}, N775$^{5.32}$, N860$^{7.46}$ of ADGRG2. (**C**) Detailed view of the angular shifts of TM1, TM5, and TM6 between ES-4 and the

active state of ADGRG2 (PDBID: 7WUI). We quantified the extent of movement of each transmembrane helix, showing how the structure of F601E-ADGRG2 deviates from its active conformation.

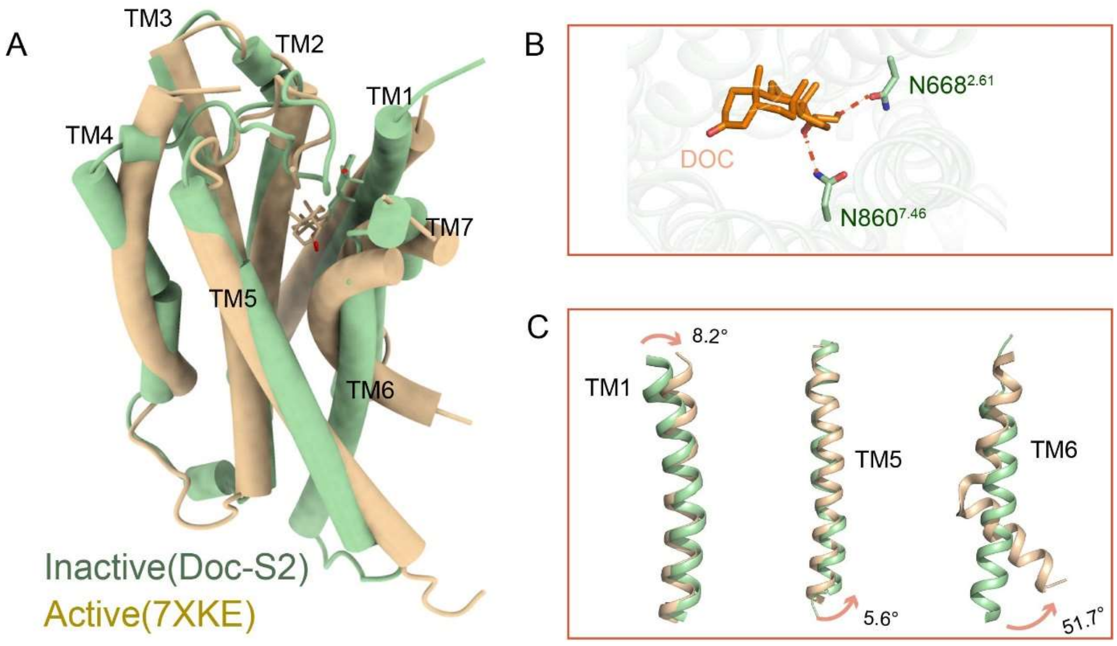

Figure S8. Doc-S2 of DOC-ADGRG2 obtained by metadynamics simulation. (**A**) Structural comparison between the inactive state Doc-S2 of DOC-ADGRG2 and the active state ADGRG2 (PDBID: 7XKE). We focus on the displacement of key transmembrane helices (TM1, TM5, and TM6) in Doc-S2 relative to the active state. (**B**) Demonstration of the polar network formed by DOC and ADGRG2. In Doc-S2 state, DOC can form a polar network with N668$^{2.61}$, N860$^{7.46}$ of ADGRG2. (**C**) Detailed view of the angular shifts of TM1, TM5, and TM6 between Doc-S2 and the active state of ADGRG2 (PDBID: 7XKE). We quantified the extent of movement of each transmembrane helix, showing how the structure of DOC-ADGRG2 deviates from its active conformation.

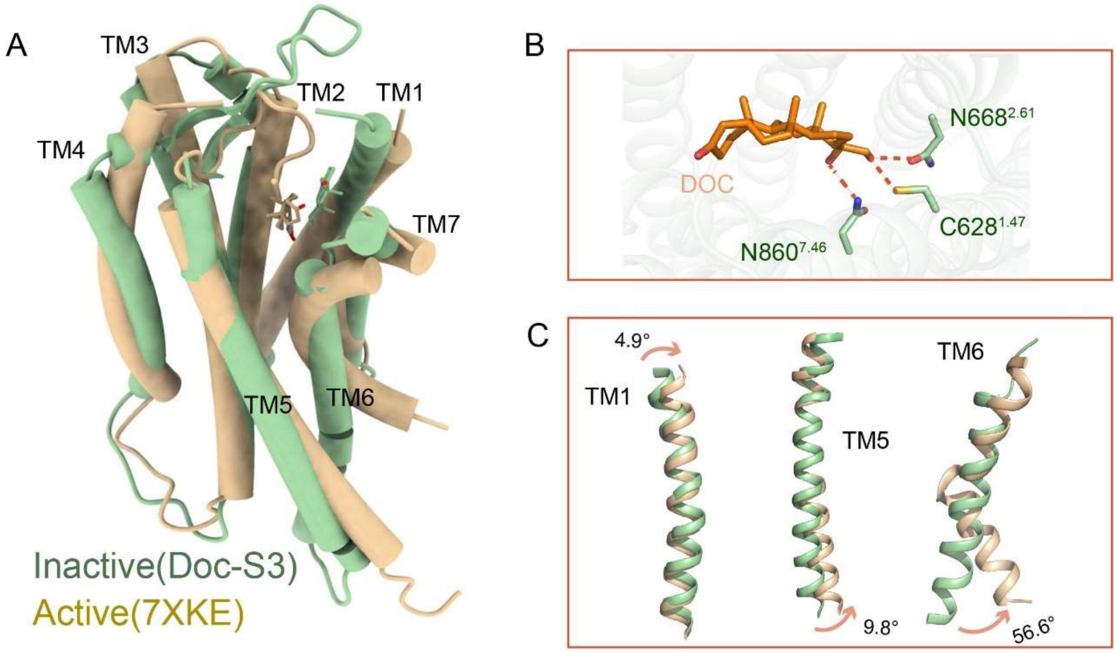

Figure S9. Doc-S3 of DOC-ADGRG2 obtained by metadynamics simulation. (**A**) Structural comparison between the inactive state Doc-S3 of DOC-ADGRG2 and the active state ADGRG2 (PDBID: 7XKE). We focus on the displacement of key transmembrane helices (TM1, TM5, and TM6) in Doc-

S3 relative to the active state. (**B**) Demonstration of the polar network formed by DOC and ADGRG2. In Doc-S3 state, DOC can form a polar network with C628$^{1.47}$, N668$^{2.61}$ and N860$^{7.46}$ of ADGRG2. (**C**) Detailed view of the angular shifts of TM1, TM5, and TM6 between Doc-S2 and the active state of ADGRG2 (PDBID: 7XKE). We quantified the extent of movement of each transmembrane helix, showing how the structure of DOC-ADGRG2 deviates from its active conformation.

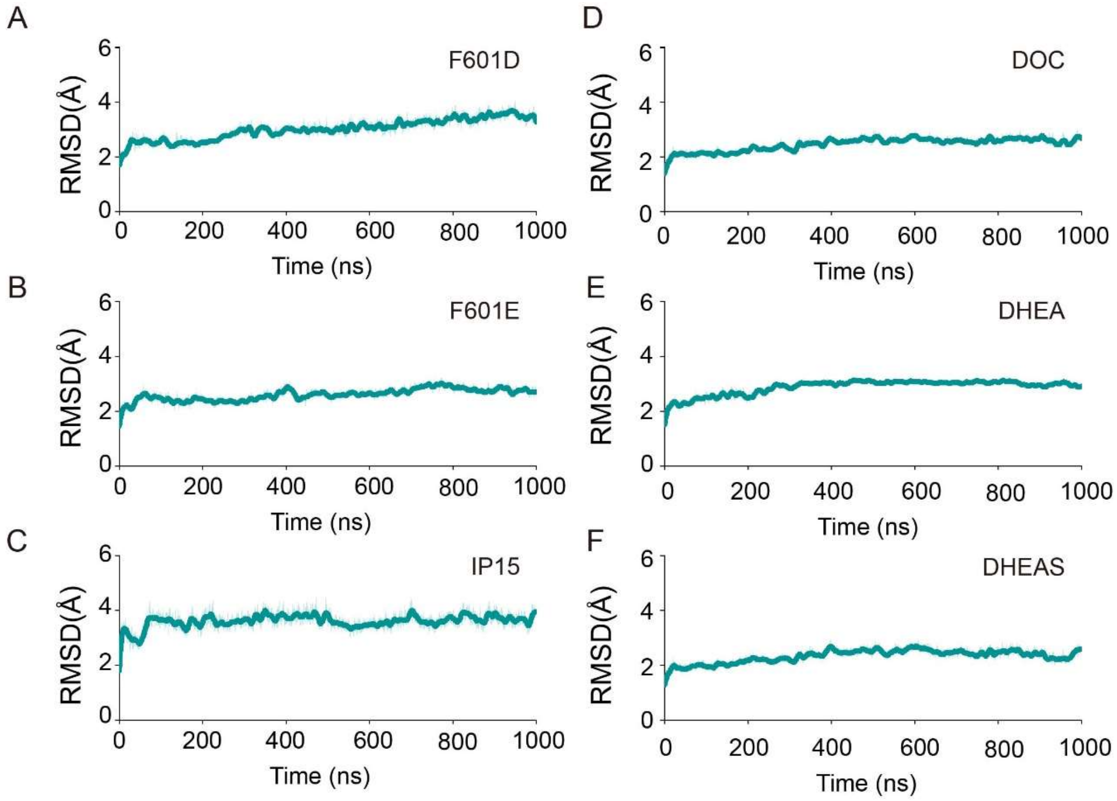

Figure S10. Simulation systems for MM-PBSA energy calculation. RMSD values for the simulation systems of F601D (**A**), F601E (**B**), IP15 (**C**), DOC (**D**), DHEA (**E**) and DHEAS (**F**). The shaded area represents the original, unsmoothed value, with reduced transparency, and the thick line was the result of smoothing the original distance value every 200 points.
